# Three-dimensional (3D) quantitative evaluation of the morphological changes of the upper anterior alveolar bone after retraction of a maxillary incisor

**DOI:** 10.1186/s12903-023-02976-2

**Published:** 2023-05-15

**Authors:** Hong Liu, Xun Xi, Dongxu Liu

**Affiliations:** Department of Orthodontics, Shandong Provincial Key Laboratory of Oral Biomedicine, School of Dentistry, Shandong University, Jinan, 250012 China

**Keywords:** Cone-beam computed tomography, Superimposition, Alveolar surface modeling, Inner remodeling

## Abstract

**Background:**

The purpose of this study was to assess morphological changes of the upper anterior alveolus after retraction of a maxillary incisor by applying three-dimensional (3D) superimposition of pretreatment (T1) and posttreatment (T2) cone-beam computed tomography (CBCT) data.

**Methods:**

The study group was comprised of 28 patients with skeletal Class II malocclusion who underwent incisor retraction. CBCT data were acquired before (T1) and after (T2) orthodontic treatment. Labial and palatal alveolar thickness were assessed at the crestal, midroot and apical levels of the retracted incisors. Following three-dimensional (3D) cranial base superimposition, we performed surface modeling and inner remodeling of the labial and palatal alveolar cortex of the maxillary incisors. Paired t-tests were used to compare T0 and T1 bone thickness and volume measurements. Comparisons between labial and palatal surface modeling, inner remodeling and outer surface modeling were performed with paired t-tests in SPSS 20.0 version.

**Results:**

We observed controlled tipping retraction of the upper incisor. After treatment, the alveolar thickness on the labial sides increased and the palatal alveolar thickness decreased. The labial cortex showed a wider range of modeling area with a larger bending height and a smaller bending angle than the palatal side. The extent of inner remodeling was more prominent than the outer surface on both the labial and palatal sides.

**Conclusions:**

Adaptive alveolar surface modeling occurred in response to incisor tipping retraction on both the lingual and labial sides although these changes occurred in an uncoordinated manner. Tipping retraction of the maxillary incisors led to a reduction in alveolar volume.

## Introduction

Orthodontically induced tooth movement is the normal result after the mechanical force was applied, the predictability of orthodontic tooth movement is of significant clinical interest. After an orthodontic load was applied on the teeth, an alteration in the strain-stress distribution within the periodontal ligament (PDL) and the surrounding bone can induce alveolar bone models and remodels [1–4]. A basic concept in orthodontics is the “Bone Traces Tooth Movement” [5], which refers to the strong correlation between orthodontic tooth movement and surrounding bone remodeling. It is often thought that alveolar bone remodeling follows orthodontic tooth movement. However, the unfavorable bone response after incisor retraction such as alveolar dehiscence and fenestration is not coherence with this simple rule.

In orthodontics, the labial/lingual position of anterior teeth is very important for those patients who desire to change facial features [6, 7]. Antero-posterior tooth movements of the maxillary anterior dental segments are conventional in orthodontic treatment. In the anterior segment, both the palatal and labial cortical plates are involved in all antero-posterior tooth movements of the maxillary anterior dental segments. The maxillary anterior region is the best model to use when investigating the relationship between bone remodeling and tooth movement [8, 9]. The retraction of incisors is a complicated process requiring changes in the periodontal ligament (PDL) as well as the supporting alveolar bone. Coordinated PDL and periosteal bone modeling allow a tooth to maintain its periodontal support while changing its position relative to the apical base. In an ideal incisor retraction, the modeling drift of the lingual and labial cortex should occur to the same extent; the balance between the labial and lingual musculature leads to healthy periodontal conditions. However, cortical bone modeling/remodeling cannot always keep pace with incisor movement and can lead to morphological alterations of the lingual and labial alveolus. Therefore, the adaptive remodeling capacity of the alveolar socket during incisor retraction has attracted significant attention with regards to orthodontic clinical studies [5, 8–11]. However, the specific modeling or remodeling characteristics of the lingual and labial cortex remain unclear.

One of the key elements to successful orthodontic treatment is the detailed evaluation of treatment outcomes. The assessment of treatment outcomes by cone beam computed tomography (CBCT) has the potential to unravel the specific interactions between the dental, skeletal and soft tissue components that underpin the response to treatment [12, 13]. Three-dimensional (3D) superimpositions can reveal areas of alveolar bone displacement and remodeling [14]. Gaining an enhanced understanding of maxillary anterior alveolar modeling/remodeling could improve our interpretations of variations in patient response to treatment. Therefore, in the present study, we investigated modeling phenomena of the alveolus from a “whole bone” perspective and evaluated the relationship of labial and lingual alveolar outer surface modeling and maxillary incisor movement with 3D superimposition on CBCT images. We also attempted to identify differences between the characteristics of labial and lingual alveolar cortex modeling during maxillary incisor retraction to provide a new understanding of alveolar bone biology.

## Materials and methods

### Study sample

This retrospective study was approved by the Biomedical Ethics Committee of Shandong Hospital of Stomatology and all subjects provided signed and informed consent prior to the study. We analyzed 3D CBCT radiographs taken before (T1) and after orthodontic treatment (T2). Data was obtained from 28 non-growing patients at the orthodontic clinic of Shandong University. DICOM files from each patient were evaluated using MIMICS software, version 21.0 (Materialise, Leuven, Belgium). To eliminate inter-examiner error, one examiner (HL) registered and measured all images. To reduce intra-examiner error, all superimpositions and measurements were performed at least three times and mean values were recorded and analyzed.

Images were taken from patients who were selected according to the following criteria: (1) non-growing adult patients; (2) class II division 1 malocclusion (5°>ANB > 3°) with increased inclination of the maxillary incisor with incisor retraction distances ranging from 4 to 6 mm; (3) mean mandibular plane angles of 25.6° ± 5.6°; (4) anterior crowding ≤ 3 mm; (5) no periodontal disease and no caries, endodontic treatment of the maxillary incisor; no obvious root resorption, no dehiscence or fenestration before treatment and (6) the dental casts, cephalograms and CBCT before and after treatment of patients were complete.

All patients were treated by a straight wire orthodontic technique after extraction of the 4 first premolars with sliding mechanics for anterior teeth retraction and space closure. The appliances for en-masse anterior retraction were 0.019 × 0.025-inch stainless steel basal archwires with incisor lingual root torque and four elastic chains. Class II intermaxillary elastics (prescribed to be worn full-time, 24 h/day) were applied for correcting the molar relationship.

#### Segmentation and reconstruction

**Fig. 1:**
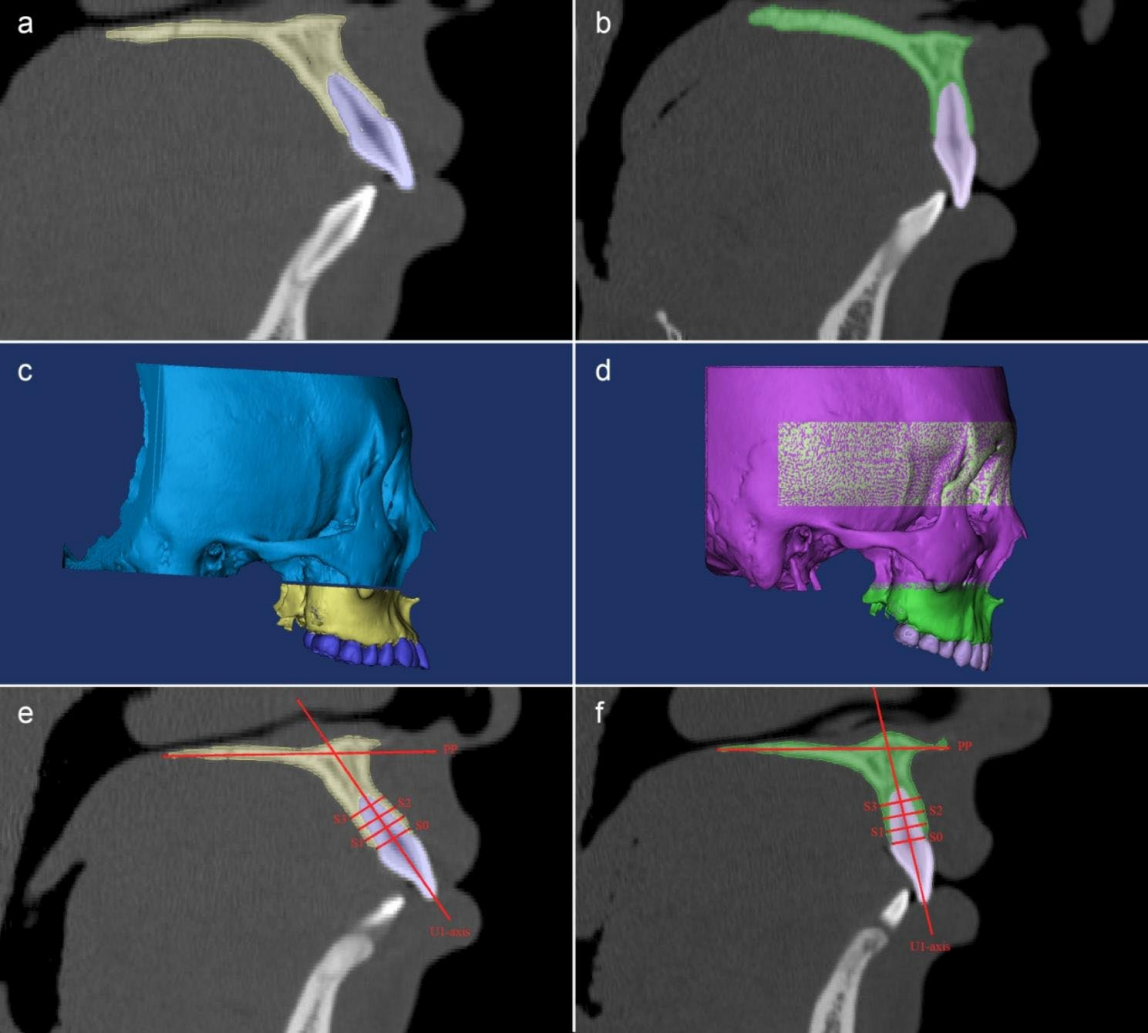
The 3D segmentation and reconstruction of T1 and T2 CBCT images. The maxillary teeth, maxillary alveolar bone and cranial base of T1 **(a, c)** and T2 **(b, d)** were reconstructed with various threshold values. In the U1-axis sagittal plane, the labial, palatal and total alveolar bone thicknesses were measured in three slices separated by 3 mm (S1, S2 and S3, respectively). We also assessed the U1-PP angles of T1 (e) and T2 (f)

Image segmentation (Fig. 1a-b) of the interested anatomic structures and 3D graphic rendering was performed by using the Mimics medical imaging density segmentation software 21.0. All 3D models of T1 and T2, such as the maxillia, the cranial base and the upper teeth were reconstructed respectively (Fig. 1c-d). The paired T1 and T2 models were exported for following superimposition.

#### 3D superimposition basing on the cranial base

**Fig. 2:**
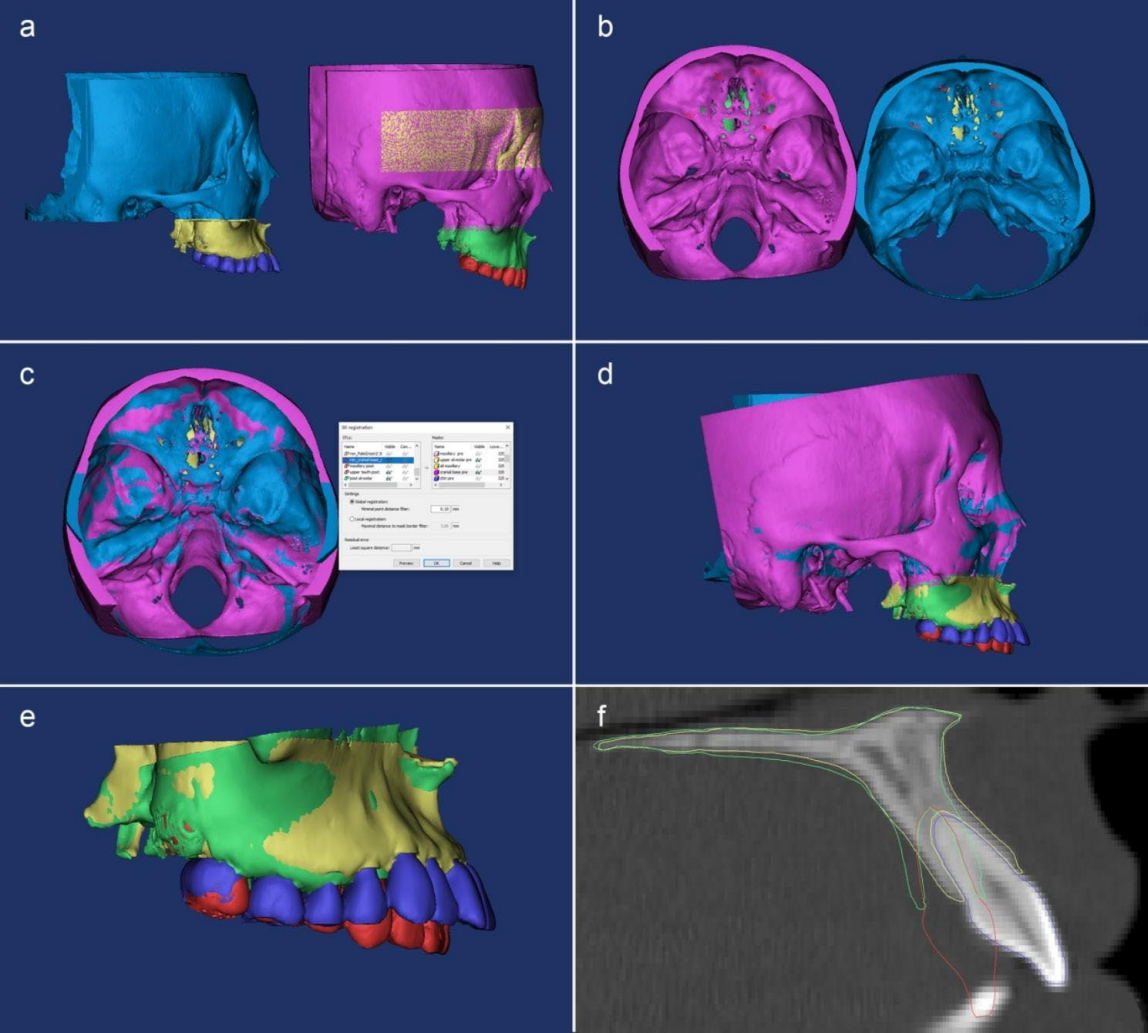
Superimposition of T1 and T2 models with the cranial base. The T2 cranial base, maxillary alveolar bone and teeth were imported into T1 project **(a)**; the superimpositions were performed with point registration **(b**) and STL registration **(c)** independently by using the anterior cranial fossa as the reference. The T1 and T2 projects were aligned in the same coordinate system **(d)** and the maxillary alveolar bone and teeth were aligned together for calculating positional and morphological changes (e, f)

In Mimics software, the 3D models of postreatment (T2) were registered to the T1 structures with the anterior cranial fossa as the reference by using point registration and STL registration tool. The endocranial surfaces of the cribriform plate region of the ethmoid bone and the frontal bone were chosen for the location of reference points and STL registration (Fig. 2). These regions were chosen because of their early completion of growth and their continuous stability [15].

Superimposition was performed initially by manual point registration to approximate the surfaces as much as possible. The cranial surface feature points which were easy to identify on the anterior cranial fossa were located for initial superimposition (Fig. 2a-b). The T1 maxillary and cranial base were registered to the T2 models in a gross manner. Subsequently, superimposition was refined using the Standard Tessellation Language (STL) registration method. The transformation matrix of this method registered the stack of slices for the inferior chin STL model on the stack of slices of the T2 cranial base mask sequentially (Fig. 2c, d). These transformations of the 3D models (cranial base, maxilla and upper teeth) were synchronous. Following sequential rigid superimpositions, the changes between the T1 and T2 maxillary anterior alveolus can be assessed (Fig. 2e, f).

#### Measurement of alveolar changes

To evaluate changes in the alveolar bone of the central incisors, most of the linear and angular measurements were performed on sagittal planes that passed through the axis of the central incisors. The parameters measured in this study included incisor movement, alveolar bone thickness changes, labial/lingual surface modeling and inner PDL remodeling.

**Fig. 3:**
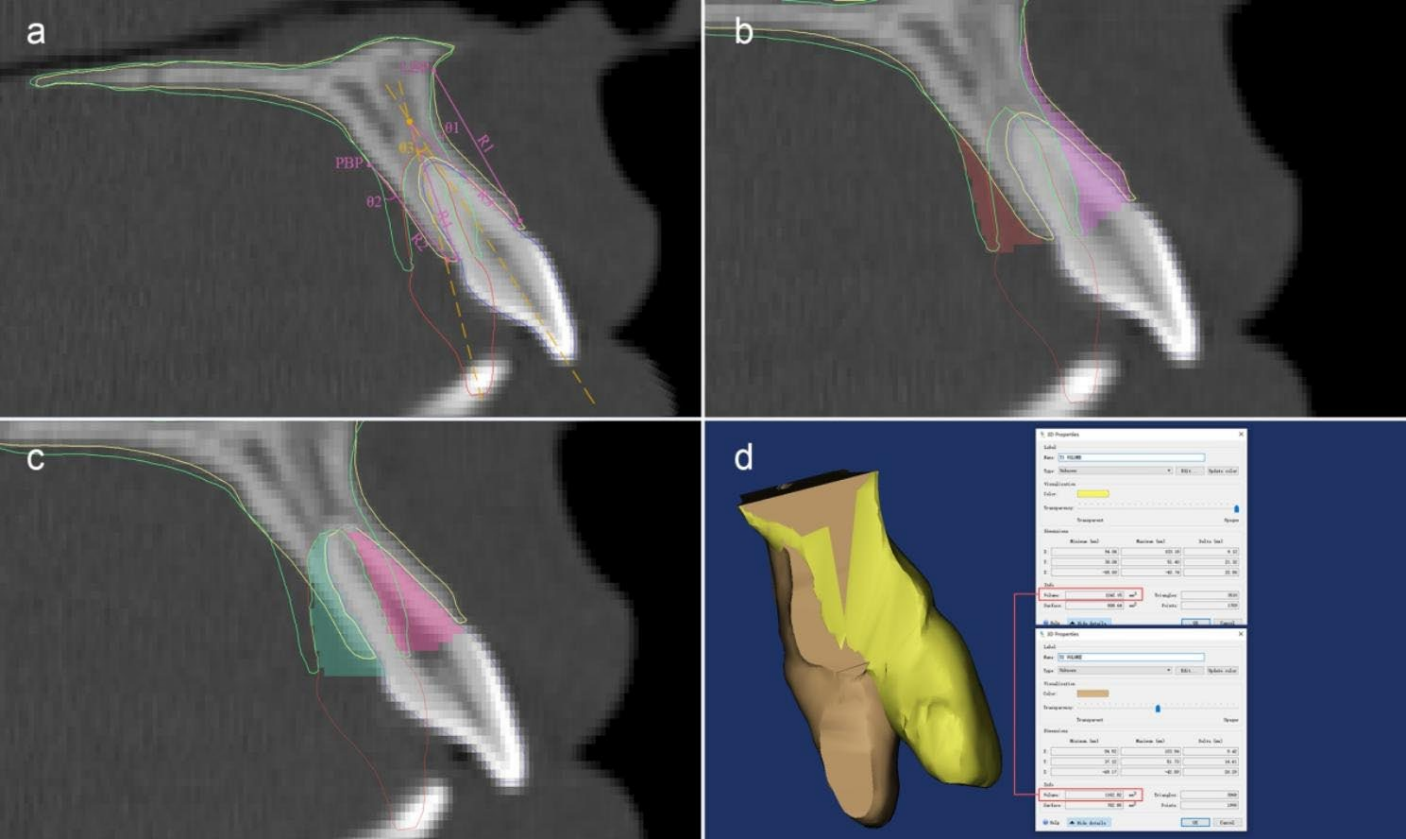
Assessment of the morphological changes of alveolar bone surrounding the central incisor. **(a)** the linear and angular measurement parameters, including the bending points for the labial and palatal cortex (LBP and PBP), the bending angle (θ1, θ2 and θ3) and the bending height (R1-R4). The surface modeling area (A1, A2) and inner remodeling area (A3, A4) of the labial and palatal alveolar cortex were also calculated **(b, c)**. After registration, we cut the alveolar bone of the central incisors at the labial bending point level and then calculated the volumes of T1 and T2 alveolar bone surrounding the central incisors for subsequent comparison **(d)**

**Fig. 4:**
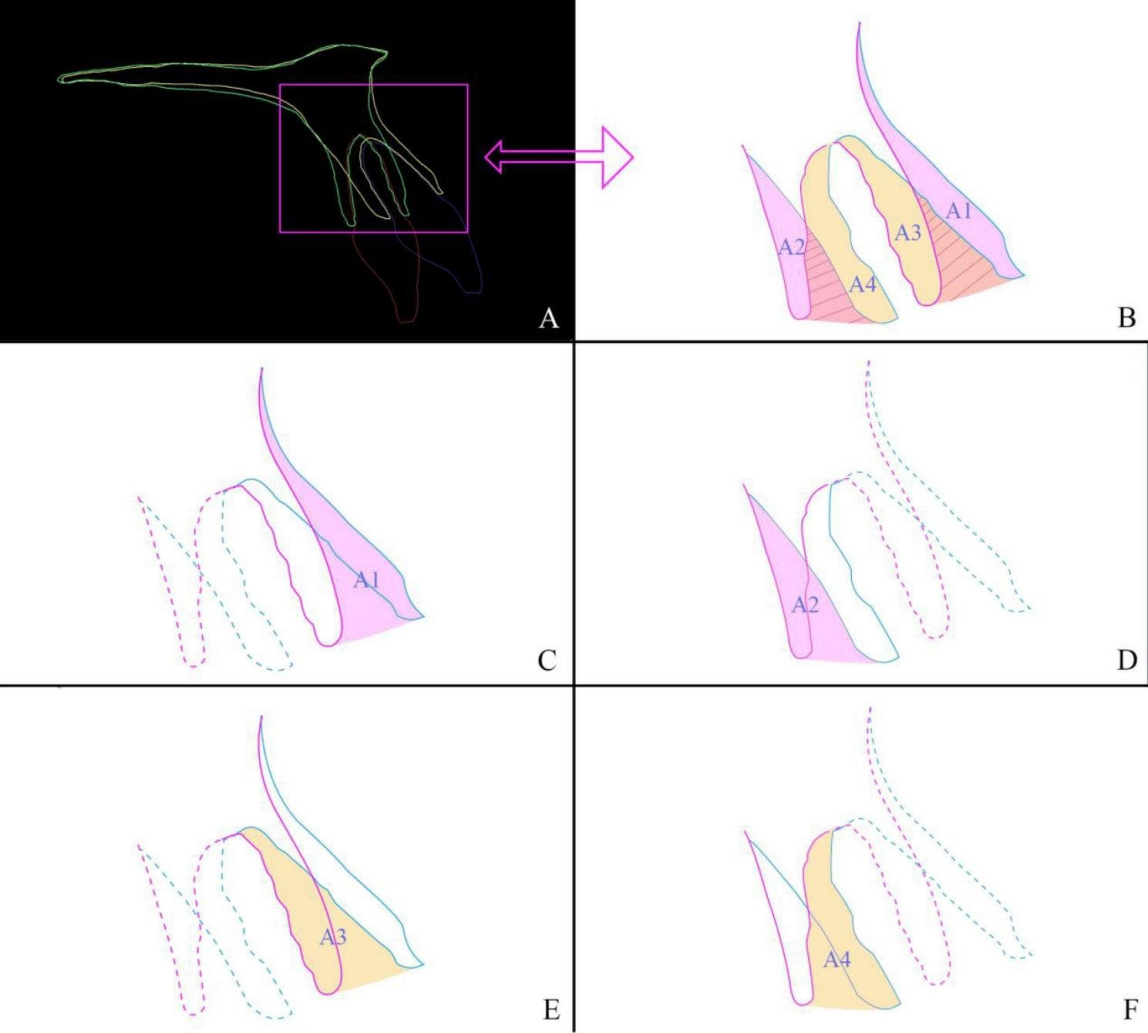
A simple diagram describing alveolar modeling/remodeling. When an incisor was retracted, the bending of the labial and palatal cortex followed the direction of the incisor movement, thus leading to alveolar surface modeling and inner PDL remodeling **(a)**. The areas of alveolar surface modeling and inner PDL remodeling were measured **(b)**. Paired comparisons between A1 and A2 **(c, d)**, A1 and A3 **(c, e)**, A2 and A4 **(d, f)** were performed to analyze alveolar morphological changes

1. The thicknesses of the labial and palatal alveolar plates were measured for each central incisor in T1 and T2 project respectively. Alveolar bone thickness was measured as the distance between the root surface and the cortical plate, perpendicular to the long axis of the tooth, at 3 (S1), 6 (S2) and 9 (S3) mm from the cementoenamel junction (S0) level. The PP-U1 axis angle was also measured so that we could evaluate the controlled tipping movement of the upper central incisors during retraction (Fig. 1e, f).
2. In the U1-axis sagittal plane and to evaluate the alveolar bending of labial and palatal cortex, the bending heights of the labial (R1) and palatal (R2) alveolar surface were measured as the linear distance between the bending point and the Cemento-Enamel-Junction (CEJ). The labial surface bending angle (θ1) and the palatal surface bending angle (θ2) were measured as the angle between T1 and T2 in the labial/palatal cortex surface. The inner labial and palatal cortex bending point was located at the intersection point for the T1 and T2 U1 axis. The virtual labial/palatal inner bending height (R3/R4) was measured between the bending point and the CEJ. The angle between T1 and the T2 U1 axis was measured and defined as the inner bending angle (θ3). The angular and linear variables are shown in Fig. 3a.
3. Considering the vertical alveolar loss, we calculated the virtual alveolar modeling/remodeling areas (VA1-VA4) with the following formula: area = πR^2^ × θ/360.
4. Alveolar bone adaptation (area) measurement: In the U1-axis sagittal plane, we measured the labial/palatal surface modeling areas (A1, A2) between the T1 and T2 labial/palatal surfaces with the “area tool”. We also measured the labial/palatal inner remodeling areas (A3, A4) (Fig. 3b, c). The four modeling and remodeling areas represent the adaptive responses of the maxillary anterior alveolus and clarify alveolar morphological changes (Fig. 4).
5. Segmentation the alveolar bone surrounding the central incisor was performed at the labial bending point level after superimposition; the T1 and T2 total volumes of alveolar bone were then measured and compared (Fig. 3d). All variables are named and described in Table 1.

**Table 1:** Key measurement parameters for alveolar modeling and remodeling

Measurement parameters	Definition
Outer labial cortex bending height (R1)	Distance from the labial bending point to CEJ
Labial cortex bending angle (θ1)	Angle of T1 and T2 labial surface contour line
Labial cortex surface modeling (A1)	The area between T1 and T2 labial surface
Virtual labial cortex surface modeling (VA1)	π (R1)^2^ × θ1/360
Outer palatal cortex bending height (R2)	Distance from the palatal bending point to CEJ
Palatal cortex bending angle (θ2)	Angle of T1 and T2 palatal surface contour line
Palatal cortex surface modeling (A2)	The area between T1 and T2 palatal surface
Virtual palatal cortex surface modeling (VA2)	π (R2)^2^ × θ2/360
Labial inner cortex remodeling (A3)	The area between T1 and T2 labial inner cortical surface
Palatal inner cortex remodeling (A4)	The area between T1 and T2 palatal inner cortical surface
Inner alveolar bending angle (θ3)	Axial angle of T1 and T2 central incisors
Inner labial cortex bending height (R3)	Distance from axial intersection point of T1 and T2 central incisors to T1 labial CEJ
Virtual inner labial cortex modeling (VA3)	π (R3)^2^ × θ3/360
Inner palatal cortex bending height (R4)	Distance from axial intersection point of T1 and T2 central incisors to T1 palatal CEJ
Virtual inner labial cortex modeling (VA4)	π (R4)^2^ × θ3/360

## Statistical analysis

For all measurements, computer-aided descriptive statistical analysis was performed using SPSS version 20.0 (SPSS Inc., Chicago, IL, USA). The mean and standard deviation (SD) of each variable measurement were estimated. Differences in central incisor movement and changes in the alveolar bone between T1 and T2 were evaluated by using the paired t-test. In addition, paired t tests were used to evaluate labial and palatal surface modeling. Comparisons between inner remodeling and outer modeling of labial/palatal cortex were performed with the paired t test. The level of significance was set at P < 0.05.

To reduce measurement error, one month after the first measurements, ten cases were randomly selected and remeasured by the same examiner (HL). Differences between the measured and mean values were used to determine the method error according to Dahlberg’s formula (δ^2^ = Σd^2^/2n) [16]. Error estimations using the method error according to Dahlberg fell below the reference value of 1.0 for all measured values.

## Results

Each patient was evaluated with a focus on alveolar morphological changes of the central incisors from the prospective of cranial base superimposition. No statistically significant differences between the right and left sides were observed.

The U1-PP angle showed an average controlled tipping movement. Morphological variables such as labial/palatal alveolar thickness, total volume of the U1 alveolus were measured and recorded in Table 2. There were statistically significant differences between T1 and T2 morphological variables. The alveolar thickness in the labial sides increased and the palatal alveolar thickness decreased. The total volume and thickness of the alveolus surrounding the central incisors were reduced after retraction. Analysis indicated that outer periosteal modeling did not keep pace with the inner PDL remodeling and that the palatal surface apposition and labial surface resorption were not synchronized and coordinated.

The results of paired t-tests for labial and palatal alveolar surface modeling variables are shown in Table 3. We did not observe root resorption and alveolar bone loss; palatal alveolar vertical bone loss was more common than on the labial side and there was some error in the measurement of palatal modeling. In addition to the actual measurement of modeling areas, the virtual modeling area was also calculated using a specific formula (area = π R^2^ × θ/360). There were significant differences between labial and palatal modeling; the labial cortex showed a wider range of modeling with a larger bending height and a smaller bending angle. The labial virtual and actual alveolar modeling areas were larger than on the palatal side. The palatal alveolar modeling was delimited with a smaller range and extent. The non-coordination between labial resorption and palatal apposition reduced the total alveolar volume surrounding the central incisor.

Comparisons between inner and outer alveolar cortex modeling are shown in Table 4. Inner alveolar remodeling and touter surface modeling were compared for both the labial and palatal sides. There were significant differences between the inner and outer alveolar modeling areas on the palatal side. The resorption modeling area on the PDL side was more profound than the palatal surface apposition; this induced the loss of palatal alveolar bone. On the labial side, the extent of labial surface resorption was less than the inner PDL apposition; the thickness of the labial alveolar cortex increased slightly.

**Table 2:** Paired t-test of alveolar bone thickness/volume and PP-U1 angle

Variables	T1	T2	P Value
Alveolar volume (mm^3^)	1297.5 ± 128.4	1076.4 ± 166.9	*
Total alveolar thickness (mm)			
S1	8.34 ± 0.65	7.91 ± 0.55	*
S2	8.97 ± 0.92	8.01 ± 0.63	*
S3	9.08 ± 0.89	8.30 ± 0.59	*
Labial alveolar thickness (mm)			
S1	1.23 ± 0.31	1.35 ± 0.36	*
S2	1.37 ± 0.40	1.48 ± 0.48	*
S3	1.65 ± 0.41	1.68 ± 0.39	NS
Palatal alveolar thickness (mm)			
S1	1.34 ± 0.26	1.06 ± 0.21	*
S2	2.17 ± 0.49	1.61 ± 0.37	*
S3	2.98 ± 0.58	2.15 ± 0.42	*
PP-U1 axis (degree)	124 ± 4.5	115 ± 4.9	*

Values are presented as mean ± standard deviation.

*=P < 0.05

NS: No significant differences.

**Table 3:** Paired t-test of labial and palatal alveolar surface modeling area

Variables	Labial	Palatal	P Value
Bending height (mm) R	14.58 ± 1.32	9.76 ± 1.02	*
Bending angle (degree) θ	10.27 ± 1.20	14.65 ± 1.35	*
Surface modeling area (mm2) A	22.10 ± 2.35	15.42 ± 2.67	*
Virtual modeling area (mm2) VA	23.95 ± 2.04	19.45 ± 1.44	*

Values are presented as mean ± standard deviation.

*=P < 0.05

**Table 4:** Paired t-test of alveolar surface modeling and inner PDL remodeling

Variables	Inner	outer	P Value
Labial cortex			
Bending height (mm) R	12.01 ± 1.49	14.58 ± 1.32	*
Bending angle (degree) θ	11.82 ± 1.65	10.27 ± 1.20	*
Surface modeling area (mm2) A	30.51 ± 3.79	22.1 ± 2.35	*
Virtual modeling area (mm2) VA	32.44 ± 4.02	23.9 ± 2.04	*
Palatal cortex			
Bending height (mm) R	12.28 ± 1.55	9.76 ± 1.02	*
Bending angle (degree) θ	11.82 ± 1.65	14.65 ± 1.35	*
Surface modeling area (mm2) A	24.14 ± 4.18	15.42 ± 2.67	*
Virtual modeling area (mm2) VA	33.01 ± 3.69	19.45 ± 1.44	*

Values are presented as mean ± standard deviation.

*=P < 0.05

## Discussion

During orthodontic treatment, the mechanical force applied to each tooth will induce an alveolar bone reaction. The mechanic force applied will move the tooth orthodontically and continue to the entire tissue, thus inducing the remodeling process. Orthodontic force will also result in alterations in the regulation of alveolar bone [17–19]. In this study, the labial and palatal morphological changes of the alveolus were evaluated quantitatively; we identified the phenomenon of coupled modeling on the labial and palatal alveolar surface. The anterior alveolar bone moved in a manner that coincided with incisor displacement. The movement of the anterior alveolar bone during incisor retraction followed the direction of incisor movement but did not remodel to the same extent; furthermore, the shape and size of the anterior alveolar bone underwent a change. Orthodontic incisor retraction is dependent upon remodeling of the periodontal ligament and the modeling of alveolar bone by mechanical means. Because the labial and lingual alveolar surface modeling followed the direction of incisor retraction, our results disputed the basic axiom of “bone traces tooth movement”. PDL remodeling in the alveolus did not trigger the synchronized and coordinated modeling changes on the alveolar outer surfaces. In this study, we observed a bending phenomenon of the alveolar bone during incisor retraction; the orthodontic force induced both PDL remodeling and alveolar modeling. To understand this alveolar response, it is necessary to consider a new theory that extends beyond the ligament to a “whole bone” perspective [20].

Several different hypotheses have been suggested regarding the biomechanical nature of orthodontic tooth movement. One of the oldest hypotheses is the ‘‘pressure tension hypothesis’’ [2, 3]. The pressure-tension hypothesis assumes that displacement of the tooth in its socket compresses the alveolar bone in that direction and that this bone is then resorbed, and new bone is formed on the opposite side. This theory can elucidate the alveolar remodeling of the PDL socket, and several cytological experiments have demonstrated the pressure tension hypothesis. The second hypothesis regarding orthodontic tooth movement is the ‘‘alveolar bending hypothesis’’ which suggests that as well as deforming the PDL, tooth movement also causes deformation of the alveolar bone [21, 22]. This theory states that the bone on the side to which the tooth is pushed is bent away from the tooth and the bone on the other side is pulled towards the tooth. This theory clarified the alveolar morphological changes from a mechanical viewpoint and can elucidate alveolar remodeling of the periosteal envelop. Recently, a third hypothesis has been suggested by Melsen^3^ which intended to match orthodontic tooth movement with orthopedic bone remodeling in accordance with Frost’s mechanostat theory [23], in which low strain is considered to lead to bone resorption while high strain leads to bone formation. These three theories explain alveolar bone remodeling from different perspectives and without conflicts. To fully understand the alveolar bone biological responses to lingual orthodontic loading on the incisors, a synthetic theory should be advocated that combines the pressure tension hypothesis, alveolar bending hypothesis and Frost’s mechanostat theory.

According to Wolff’s law and Frost’s mechanostat theory, bone modeling activity is controlled by peak strain during dynamic loading [17, 24, 25]. The rate of subperiosteal bone formation is directly related to the level of surface strain [26]. The mechanostat theory may be used to explain why orthodontic forces stimulate different periosteal responses in the labial and lingual alveolar surfaces. When a labial-lingual orthodontic force was applied on the incisor, the walls of the tooth socket behave like cantilever beams; that is, they are essentially fixed at one end (towards the apex) and free at the other (towards the tooth crown). When an orthodontic load is applied, this displaces the free end and, since the other end is fixed, a slight bending of the tooth socket walls occurs. We identified different changes in stress/strain on the compressive and tension side. On the compressive side, the PDL suffered pressure and then induced a resorption response on the inner alveolar bone. As the alveolar process is thinned by bone resorption on the PDL surface, the outer plate of bone is exposed to excessive functional strain (exceeding 2500 microstrain), the pressure-induced mechanical stretch at the outer surface then induces periosteal proliferation and bone anabolic modeling. On the tension side, stretching of the PDL induces formation of the inner alveolar bone and this inner stretching results in the periosteal surface staying below the minimum effective strain (MES) as defined by Frost. As the alveolar process is thickened by bone apposition on the PDL surface, the periosteal surface is exposed to an inadequate functional strain (< 50 to 200 microstrain). Catabolic modeling is triggered by an atrophic mechanism. The alveolar process is thinned until the surface strain on the resorbing surface returns to the optimal physiological range (200 to 2500 microstrain).

The periodontal ligament is always considered as an internal alveolar periosteum; indeed, researchers consider these two structures homologous. When an orthodontic force is applied on the incisor and induces a labial-lingual movement, the alveolar bone response is a coupled process involving periodontal ligament remodeling and periosteal modeling in a manner that is similar to modeling of the periosteum and endosteum [27]. The responses of the internal and outer alveolar periosteum to incisor movement were not synchronous and the thickness of the labial and lingual alveolus underwent changes. Unlike direct loading on the PDL with greater strain, the subperiosteal modeling was driven by the indirect transmission of force through the alveolar wall and occurred in a restricted area. When the incisor was retracted, the thickness of the lingual alveolus was reduced and the thickness of the labial alveolus was increased. This non-coordinated PDL and periosteal bone modeling resulted in changes in alveolar thickness which has been reported extensively in previous studies [28].

Maxillary anterior alveolar surface modeling is an uncoupled process during incisor retraction; this means that anabolic and catabolic sites are controlled independently with different activities and rates [17]. The modeling/bending response of the lingual cortex is known to be limited to the apical region while labial modeling affected the entire anterior alveolar wall. In the present study, we identified significant differences between the dimensions of the labial and palatal alveolar wall. The labial wall on the maxillary anterior teeth was predominantly thin; most measurements were less than 1 mm. The thickness of the palatal wall was significantly larger than that of the labial wall. From the alveolar crest to the apical root, the labial alveolar thickness was uniform while the lingual alveolar thickness increased significantly. It is difficult for the strain on the palatal alveolar surface to exceed the physiological range due to the dentoalveolar anatomy of the palatal alveolar wall. Furthermore, palatal subperiosteal modeling cannot be triggered unless the root of the incisor is close to the lingual cortex. Considering the difference between the labial and lingual alveolar cortex of the maxillary incisors, non-harmonious maxillary anterior alveolar bone modeling may be inevitable during incisor retraction. Anabolic modeling along the periosteal surface in the direction of tooth movement is the critical process for maintaining alveolar bone support during tooth movement [29]. The restricted alveolar modeling response, due to the low mechanosensitivity and mechanotransduction of the palatal alveolar surface, may decentralize teeth from the alveolar bone envelope, thus causing bone dehiscence and fenestrations and gingival recession [30].

In this 3D superimposition study, we found that labial and palatal alveolar modeling occurred during maxillary incisor retraction and that this labial and palatal surface modeling was not coordinated. Furthermore, we advocate a new synthetic theory that combines the pressure tension hypothesis, alveolar bending hypothesis and Frost’s mechanostat theory. To confirm this new hypothesis of periosteal modeling, further research needs to consider mechanotransduction, including mechanocoupling, biomechanical coupling, cell-to-cell signaling, and the response of effectors in the maxillary incisor retraction process.

## Conclusion

The adaptive alveolar surface modeling was occurred responding to the incisor tipping retraction in both lingual and labial sides with uncoordinated amount. The tipping retraction of maxillary incisor decrease the alveolar volume.

## Data Availability

The datasets used and/or analysed during the current study available from the corresponding author on reasonable request.
